# *Listeria monocytogenes*: a promising vector for tumor immunotherapy

**DOI:** 10.3389/fimmu.2023.1278011

**Published:** 2023-10-06

**Authors:** Yi-Dan Ding, Lin-Zhen Shu, Rui-Shan He, Kai-Yun Chen, Yan-Juan Deng, Zhi-Bin Zhou, Ying Xiong, Huan Deng

**Affiliations:** ^1^ Medical College, Nanchang University, Nanchang, China; ^2^ Office of Clinical Trials Administration, The Fourth Affiliated Hospital of Nanchang University, Nanchang, China; ^3^ Department of Pathology, The Fourth Affiliated Hospital of Nanchang University, Nanchang, China; ^4^ Tumor Immunology Institute, Nanchang University, Nanchang, China; ^5^ Department of General Medicine, The Second Affiliated Hospital of Nanchang University, Nanchang, China

**Keywords:** *Listeria*, immunotherapy, cancer vaccine, tumor, therapy

## Abstract

Cancer receives enduring international attention due to its extremely high morbidity and mortality. Immunotherapy, which is generally expected to overcome the limits of traditional treatments, serves as a promising direction for patients with recurrent or metastatic malignancies. Bacteria-based vectors such as *Listeria monocytogenes* take advantage of their unique characteristics, including preferential infection of host antigen presenting cells, intracellular growth within immune cells, and intercellular dissemination, to further improve the efficacy and minimize off-target effects of tailed immune treatments. *Listeria monocytogenes* can reshape the tumor microenvironment to bolster the anti-tumor effects both through the enhancement of T cells activity and a decrease in the frequency and population of immunosuppressive cells. Modified *Listeria monocytogenes* has been employed as a tool to elicit immune responses against different tumor cells. Currently, *Listeria monocytogenes* vaccine alone is insufficient to treat all patients effectively, which can be addressed if combined with other treatments, such as immune checkpoint inhibitors, reactivated adoptive cell therapy, and radiotherapy. This review summarizes the recent advances in the molecular mechanisms underlying the involvement of *Listeria monocytogenes* vaccine in anti-tumor immunity, and discusses the most concerned issues for future research.

## 1 Introduction

Cancer constitutes a significant factor for the worldwide mortality rate. In 2023, the United States will experience 1,958,310 new cancer cases and 609,820 deaths (1). Conventional treatments such as surgery, radiotherapy, and chemotherapy still suffered from recurrence, metastasis, and drug resistance, leading to an urgent need for novel therapeutic strategies (2). Therefore, tumor immunotherapy has emerged (3), with the aim to destroy tumor cells selectively via activating or reactivating host cellular immunity mainly mediated by T cells (4). Immunomodulatory drugs can also work against cancer cells through increasing the concentration of tumor-specific antibodies, natural killer (NK) cells, dendritic cells (DCs), macrophages and cytokines. Recent studies support tumor immunotherapy as an effective strategy, which can surpass the constraints of conventional treatments and improve the prognosis of patients with different malignancies (3).

However, the efficacy of immunotherapy is greatly affected by the tumor microenvironment (5). Clinically, some patients show low immune responses and limited benefits. Thus, immunotherapy based on different vectors is expected to reshape TME. Bacteria are regarded as suitable carriers due to their immunogenicity and tropism for hypoxic tissue. Bacteria-based immunotherapy can colonize TME to effectively activate the immune system of patients who do not response to conventional treatments (6).

*Listeria monocytogenes* (*Lm*), a Gram-positive bacterium, is facultatively anaerobic. *Lm*-infected host exhibits mild to severe gastroenteritis, bacterial sepsis, and even bacterial meningitis (7). The virulence of *Lm* increases in direct proportion to its colonization and dissemination ability (8). Pore-forming toxin listeriolysin-O (LLO) facilitates the escape of *Lm* from the phagosome of phagocytic and nonphagocytic cells (9). Thus, *Lm* can be found both in the cytoplasm and endosomal compartments (10, 11). Besides, *Lm* has a unique life cycle and capabilities to induce a robust cytotoxic immune cell response (12). The *Lm* surface proteins, internalin A (inlA) and internalin B (inlB), interact with surface receptors E-cadherin and C-Met to facilitate the entrance of *Lm* into nonphagocytic cells (13, 14). After internalization, *Lm* is enclosed in the host phagosome. Through secreting phospholipases (plcA and plcB) and LLO, *Lm* perforates phagosomes and enters the cytoplasm to escape phagolysosome killing (15, 16). The tumor-associated antigens (TAAs) secreted by *Lm* undergo degradation by proteasomes, subsequently stimulating specific CD8^+^ T cells via MHC class I molecules (17). Owing to hypoxia, suppressive TME, and the ability to grow intracellularly, *Lm* is easier to escape the immune surveillance and infiltrate into TME compared to other bacteria. The apparent synergy between the expansion of effector T cells and a sharp immunosuppressive cells reduction can enhance innate immunity and restructure TME to improve the efficacy of immunotherapies. Many studies have shown significant interest in *Lm* and tried to make it a vector to promote tumor immunotherapy. Nevertheless, using *Lm*-based therapy alone still faces the problem of poor therapeutic effects and complications because of its potential pathogenicity. The combination of *Lm*-based therapy with other treatments has been proposed as a possible candidate to overcome the limitations. This review will focus on how *Lm*-based modulates immune pathways to hold a promising anti-tumor response and current progresses in *Lm*-based tumor immunotherapy.

## 2 Mechanisms of *Lm*-specific immunotherapy

### 2.1 An antigen vector

Cancer vaccines usually consist of TAAs and paired adjuvants. Bacteria-delivered TAAs activate specific cytotoxic T lymphocyte (CTLs) against tumor cells and induce memory T cells to prevent relapse. Both the pathway of *Lm* invasion and the ability to survive in macrophages are critical for delivering target antigens and stimulating immune responses (18, 19). Taken by oral administration or intravenous injection, *Lm* vaccines preferentially infect host antigen presenting cells (APCs) (7, 20). Internalized *Lm* can secrete LLO to enhance the permeability of the phagosome, leading to the translocation of TAAs to the cytoplasm (21). TAAs are presented by the MHC-I complex to the cell surface and activate CD8^+^ T cells (22). Moreover, *Lm* replicates and secretes actin assembly-inducing protein (actA) to polymerize host actin and facilitate its spread among cells (23, 24). These characteristics make *Lm* an attractive candidate as an antigen vector for immunotherapy.

A number of *Lm* strains expressing different antigens have been created. *Lm* can deliver prostate specific antigen (PSA) to increase CD8^+^ T cell number in spleens and tumors and inhibit regulatory T cell (Treg) allocation (25). Pancreatic ductal adenocarcinoma (PDAC) associated antigen Annexin A2 (ANXA2)-expressing *Lm* could activate CD8^+^ T cells in TME and improve the survival of the rodent models of PDAC (26). Additionally, a fusion of *Lm* antigens and TAAs enhances anti-tumor responses. Given the ability to perforate phagosomes and help *Lm* spread from cell to cell, modified LLO and actA are mostly employed as fusion partners. Although the human papillomavirus (HPV) antigen E7-expressing strain alone has almost no impact on tumor growth, the combination of *Lm*-derived E7 and truncated version LLO (tLLO) results in tumor regression in up to 75% of patients (27). Similar to tLLO, the fusion of truncated actA with E7 also shows enhanced anti-tumor effects (28).

### 2.2 Effects on immune cells in TME

**Figure 1:**
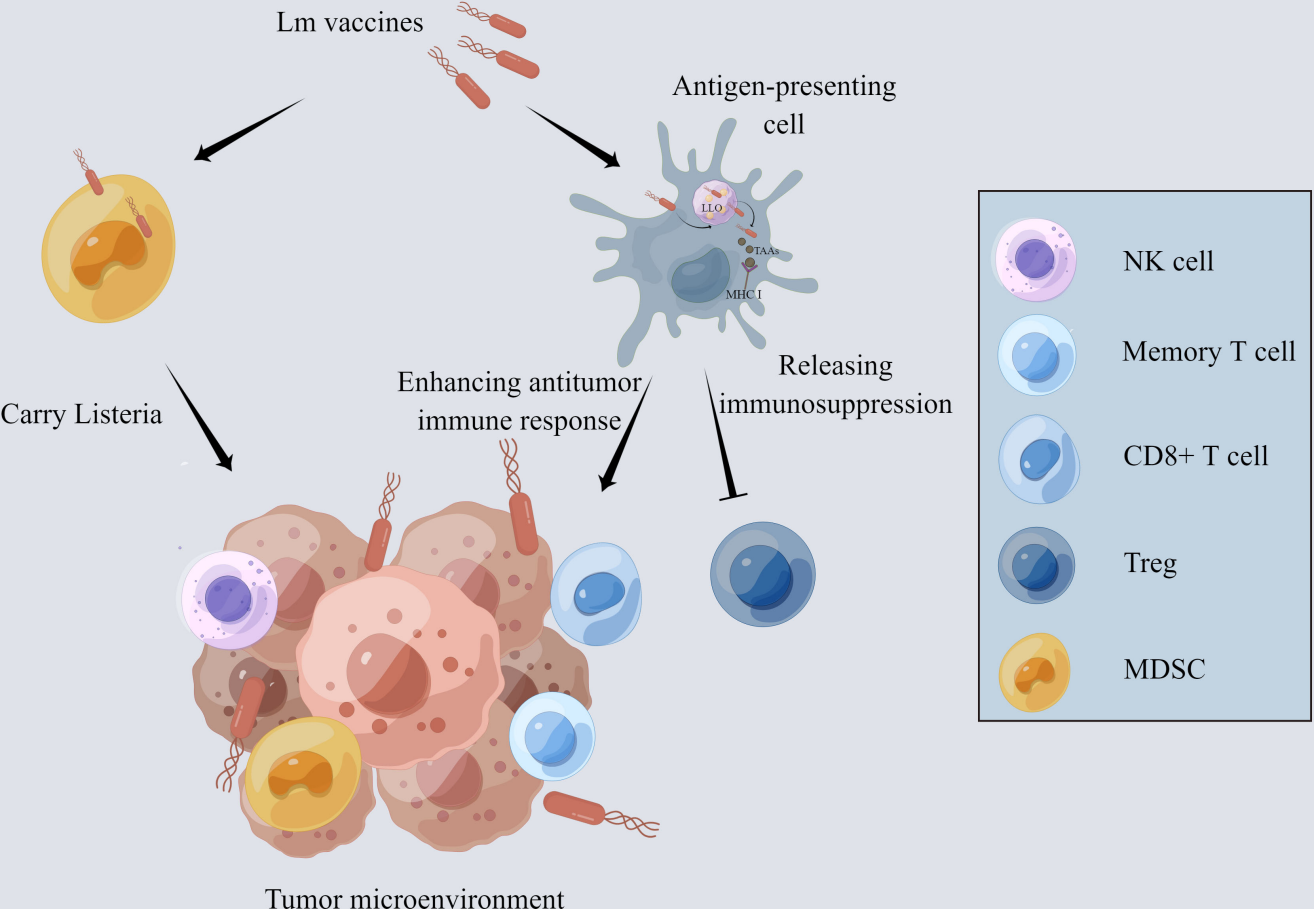
The mechanism of *Lm*-based vaccine affecting tumor microenvironment. The *Lm*-based vaccine is phagocytosed by antigen-presenting cells and escapes the phagosome by releasing the pore-forming toxin listeriolysin-O. MHCI takes up the tumor-associated antigens delivered by *Lm*-based vaccine and presents it on the cell surface, which activates the anti-tumor immune response of CD8^+^ T cells. In addition, the number and activity of memory T cells and NK cells are increased while decreasing the number and activity of Treg cells and reducing the immunosuppression in tumor microenvironment. *Lm*-based vaccine also directly infects MDSCs and spread to the TME with the aid of MDSCs.

One of the main reasons why many immunotherapies fail to provide therapeutic benefit is that the immunosuppressive microenvironment impedes anti-tumor responses. Tregs and myeloid-derived suppressor cells (MDSCs) in TME are primarily responsible for T cell inhibition and rapid depletion of tumor-specific T cells (29–31). The innate immune responses to *Lm*, including the effects of NK cells, tumor-associated macrophages (TAMs), mast cells, and neutrophils, contribute to TME remodeling (**Figure 1**).

Recent findings indicate that *Lm* vaccines promote the infiltration of IFN-γ-producing effector CD8^+^ T cells and a remarkable decrease in the frequency of FoxP3^+^ Tregs, transforming the immunosuppressive TME into inflamed and leading to the inhibition of tumor development (32, 33). Additionally, interferon-stimulated gene 15 (ISG15) exerts a key function in innate immunity. *Lm*- LLO-ISG15 induces the production of interferon-γ, attracting effector T cells to TME (34). Besides, *Lm*-based vaccines can also induce tumor-associated macrophage polarization from M2 to M1 with anti-tumor phenotype (35). *Lm* infection activates neutrophils and mast cells, which, in turn, secret IL-6 and IL-13 to enhance the host immune response (36, 37).

Moreover, *Lm* vaccines can increase the number and activity of NK cells which perform strong anti-tumor action through targeting and lysing tumor cells (32, 33, 38). *Lm* vaccine can also reduce the number of MDSCs through direct infection both in the peripheral blood and TME (39, 40). Infected MDSCs can transform into immune-stimulating phenotype and secrete IL-12 to activate the CD8^+^ T cells and immune responses (41).

In addition, *Lm* vaccine can target tumor-associated blood vessels through the surface antigens of endothelial cells (42). Endoglin (CD105), a necessary glycoprotein of the TGF-β receptor complex, has been proposed as an available marker for tumor-related angiogenesis and neovascularization. Accumulating evidence supports the high efficacy of CD105-targeted *Lm* vaccine in breast cancer models through stimulating anti-angiogenic and anti-tumor immune responses (43). CD105 is expressed on both tumor cells and endothelial cells in renal cell carcinoma (RCC) (44, 45). *Lm*-LLO-CD105A encodes a CD105 antigen fragment that targets RCC tumor cells as well as tumor-related blood vessels. The vaccine can reduce the progression of RCC in subcutaneous and orthotopic models by increasing the infiltration of polyfunctional CD8^+^ and CD4^+^ T cells (46).

Although the *Lm* vaccine has shown significant effects on remodeling TME and increasing the anti-tumor effects of specific T cells, there are still a series of concerns that need to be addressed before its clinical practice. Despite the utilization of attenuated strains, components of *Lm* still can elicit the body immunity and stimulate macrophages to phagocytize the bacteria. In addition, most bacteria-based vaccines are inevitably cleared by the reticuloendothelial system before landing on the tumor, ultimately with a lower-than-expected anti-tumor efficacy.

## 3 Development of attenuated *Lm* strains

The pathogenicity of *Lm* constitutes a significant constraint for clinical applications. Therefore, the delicate balance between the safety and immunotherapeutic efficacy of the candidates is a fundamental aspect of *Lm*-based strategies.

Selectively deleting virulence genes of wild type *Lm*, including *inlB*, *actA*, alanine racemase (*dal*), and D-amino acid aminotransferase (*dat*), has been widely used to develop an ideal vector. *InlB* directly mediates the infection of nonphagocytic cells *in vitro*, while *actA* promotes a cell-to-cell spread pattern (23, 47). The *actA* (Δ*actA*) and *inlB* (Δ*inlB*) double-deleted *Lm* strain cannot be transmitted between cells and exhibit a reduced potential to infect hepatocytes directly or indirectly, but the ability to stimulate innate immunity is intact (48). Meanwhile, the eradication of Δ*actA*/Δ*inlB Lm* strain from the liver and spleen is much faster than that of a single mutant strain (49). Moreover, attenuated Δ*actA*/Δ*inlB Lm* strain shifts the phenotype of TAMs from an inhibitory (M2) to a stimulatory state (M1), leading to good prognosis in mouse model with aggressive ovarian carcinoma (48). D-alanine is an essential component for the synthesis of the mucopeptide in *Lm* cell walls, which is controlled by two important genes, *dal* and *dat* (50). After inactivating both genes, the replication of *Lm* must be dependent on exogenous D-alanine (50). The dual deficient strain acquires *dal* and *dat* genes from *Bacillus subtilis* to restart the replication within a limited degree without serious organ damage (33).

Developing killed but metabolically active (KBMA) bacteria also represent a promising pathway. Brockstedt et al. created a nucleotide excision repair gene-deleted *Lm* vaccine (KBMA *Lm*), which is highly sensitive to photochemical inactivation induced by psoralen and long-wave UV (38). The anti-tumor KBMA *Lm* stimulates effective CD4^+^ and CD8^+^ T cell responses and an increase in the number of mature DCs in colon cancer model without significantly side effects (51, 52).

*Lm* recombinase-induced intracellular death (*Lm*-RIID) is a novel vaccine platform. By inducing Cre recombinase in host cell cytosol to delete essential genes for bacterial viability, *Lm*-RIID commits suicide intracellularly. As a result, *Lm*-RIID can elicit potent anti-tumor effects without normal tissue injury. Besides, *Lm*-RIID exhibits a higher clearance rate than double-deleted *Lm* strains. The development of *Lm*-RIID marks a significant advancement in the progress of *Lm*-based vaccine (53).

ISG15 is considered as a new TAA and therapeutic target for colorectal cancer. *Lm*-LLO-ISG15 mediates CD4^+^ T and CD8^+^ T cell responses through a greater ratio of effector to regulatory T cells in TME (54). Moreover, manganese ions (Mn^2+^) have been found to promote the activation and infiltration of CD8^+^ T cells to kill tumor cells *in vivo*, while calcium ions (Ca^2+^) can regulate autophagy to facilitate the cross-presentation of antigens (55, 56). Calcium-doped manganese carbonate microspheres and LLO (Ca@MnCO (3)/LLO) are used to form a manganese-containing multimode vaccine delivery system, which facilitates antigen cross-presentation, induces proliferation of CD8^+^ T cells, and ultimately yields significant anti-tumor effects (57).

Various methods have been employed to control the virulence of *Lm* vaccine and ensure its safety. The levels of biological materials or drugs bound to the bacterial surface do not increase with the replication of vaccine, resulting in the dilution of effective concentration. Further studies could focus on the interactions between the bacterial vector and therapeutic drugs. In addition, with regard to the risk of triggering bacteremia in the host, it would be remarkably cautious to the complications of *Lm*. In conclusion, how to ensure the maximum efficacy of *Lm* vaccines at a safe dose is still the core issue in current vaccine development.

## 4 Synergy with other therapies

**Figure 2:**
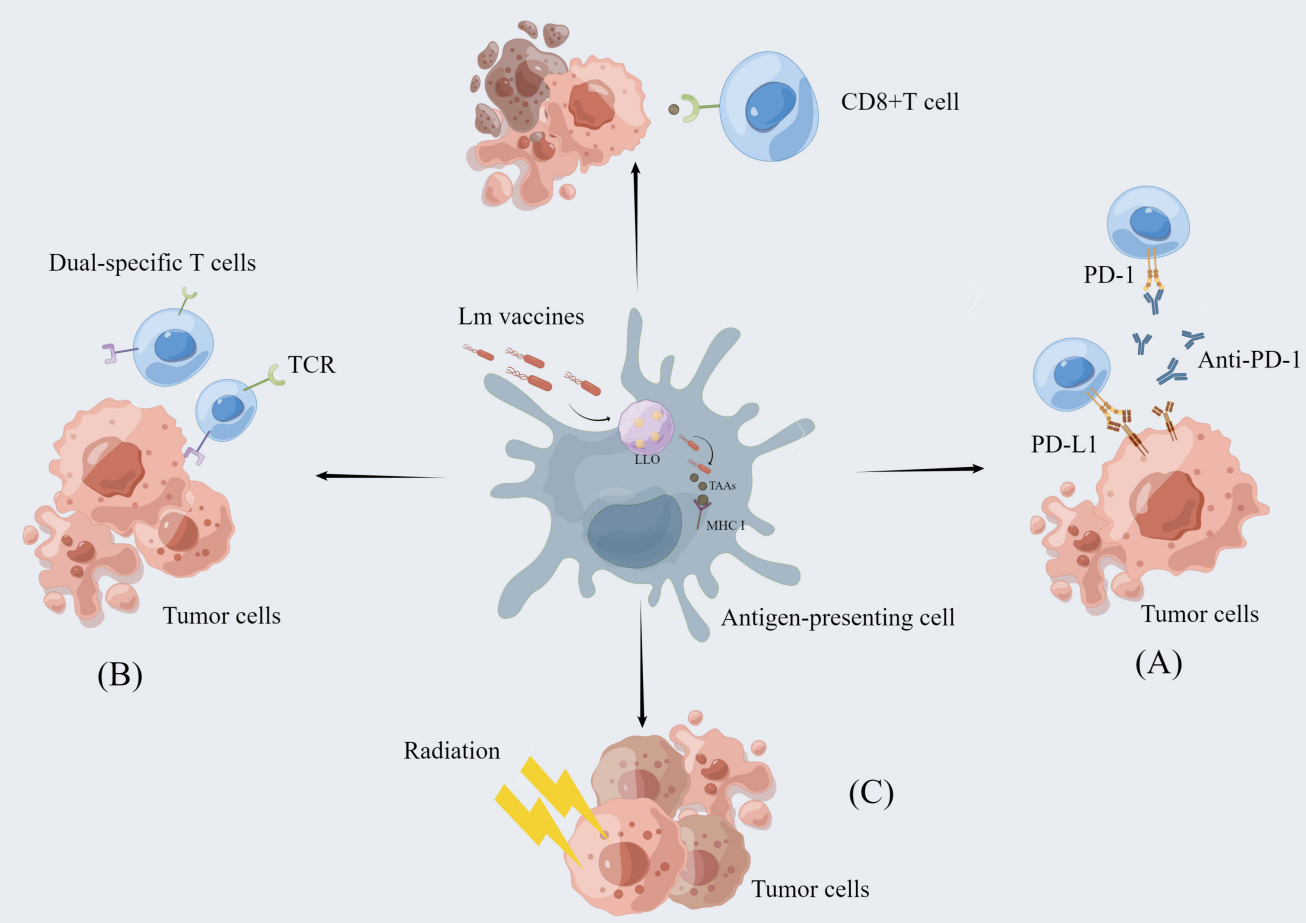
The combination of *Lm*-based vaccine and other therapies. *Lm*-based vaccines can be combined with multiple therapies to realize more potent anti-tumor effects. **(A)** Combination with immune checkpoint inhibitor therapy: Immune checkpoint inhibitors enhance the anti-tumor response of T cells by blocking the binding of checkpoint proteins to partner proteins, such as blocking the binding of PD-1 to PD-L1. *Lm*-based vaccines reduce immunosuppression by remodeling the tumor microenvironment while activating CD8+ T-cell immune responses to exert synergistic antitumor effects with ICI. **(B)** Re-energized Adoptive Cell Therapy: The ability of *Lm*-based vaccines to reduce the immunosuppressive environment in combination with the dual-specific T cells are tumor-reactive CD8^+^ T cells enhances tumor infiltration and function of CD8+ T cells. **(C)** Combined application with radiotherapy: Radiotherapy can directly cause tumor cell death and *Lm*-based vaccine can retard tumor growth. The combination of the two therapies exerts synergistic anti-tumor effect.

It has been established that carcinogenesis is largely the result of mutations in multiple genes with strong individual differences. Although *Lm*-based vaccines have shown regulatory effects on cancer immunity, not all patients can benefit from a single cancer vaccine. Thus, *Lm*-based vaccines are also used in combination with immune checkpoint inhibitors, reactivated adoptive cell therapy, and radiotherapy to achieve greater efficacy with lower side effects (**Figure 2**) (**Table 1**).

**Table 1:** The summary of *Lm*-based vaccines used in tumors.

Cancer	TAAs	*Lm* vaccine strain (reference)	Combination therapy (reference)
HPV-associated cervical cancers	HPV 16 E7	ADXS11-001 (58)	None
		*Lm*-LLO-E7	anti-PD-1 blockade (27, 39)
	E7	LM4Δ*hly*::E7-1 (59)	None
	HPV16 E6E7	LMΔ-E6E7 (11)	
Melanoma	HMW-MAA	*Lm*-LLO-HMW-MAA-C (60)	
		*Lm* ^at^-LLO (61)	
		*Lm*-OVA (62)	RT (63)
			ReACT (64)
Pancreatic cancer	TT (856–1313)	*Lm*-TT *(856*–1313) (40)	None
	ANXA2	*Lm*-ANXA2 (26)	anti-PD-1 blockade (26)
Breast cancer	position 311-660	LM-LLO-Mage-b/2^nd^ (65)	None
	Mage-b	LM-Mb (66)	
Prostate cancer	tLLO-PSA	ADXS31-142 (67)	RT (68)
	prostatic acid phosphatase, prostate-specific membrane antigen, synovial sarcoma X breakpoint 2, and homeobox protein NKX3.1	JNJ-809 (69)	None
Malignant pleural mesothelioma	mesothelin	CRS-207 (70)	PD-1 blockade (71, 72)
Hepatocellular carcinoma	fusion peptide (HBc, HBx_52-60_, HBx_140-148_, AFP_158-166_,MAGE_271-279_)	*Lm*-MPFG	anti-PD-1 blockade (35, 73)

The table provides a list of *Lm*-based vaccines for each type of cancer covered in the text, the tumor-associated antigens they target and the combination therapy with Lm-based vaccines.

### 4.1 Immune checkpoint inhibitors

As one of the most important components of the immune system, immune checkpoints can prevent healthy cells from excessive immune responses. Meanwhile, immune checkpoints can limit the potency of activated T cells through the interactions between T cells and partner proteins on target cells such as tumor cells (74). The most studied immune checkpoint molecules include cytotoxic T-lymphocyte–associated antigen 4 (CTLA-4) and programmed cell death protein-1 (PD-1) (75–78). Cancer cells express PD-L1, the ligand of PD-1, enabling the tumor to evade assault from effector T cells (79). Immune checkpoint inhibitors (ICI) refer to the utilization of blocking antibodies targeting the checkpoint molecules PD-1/PD-L1 and CTLA-4. ICI can restore the recognition and killing efficacy of immune cells and prevent the immune evasion of tumor cells (80, 81). However, separate use of ICI may not be effective for some types of cancer such as PDAC (26). The combination of blockading checkpoint protein with *Lm*-based immunotherapy has shown positive clinical effectiveness in many types of cancer, including melanoma and hepatocellular carcinoma (35, 81).

*Lm*-based vaccines can reinforce immune checkpoint blockade function to fight against cancer, mainly via ameliorating the immunosuppressive TME and inducing anti-tumor immune responses. *Lm*-LLO-E7 vaccine, formed by HPV16-E7 and truncated LLO, can enhance the efficacy of anti-PD-1 antibodies by reducing the number of Tregs and MDSCs in the spleen and TME (27, 39). ANXA2 favors the metastasis of PDAC and induces antibody responses (82). Compared with the single treatment, a combination of *Lm*-ANXA2 and anti-PD-1 antibody significantly prolonged survival time in PDAC model. Moreover, when *Lm*-ANXA2 is administered prior to the use of anti-PD-1 antibody, cure rates in implanted PDAC models are increased (26). A *Lm*-based hepatocellular carcinoma (HCC) vaccine, *ΔdalΔdat Lm*-multiple peptides fusing genes (MPFG), with the ability to secrete HCC-related TAAs fragments, activates the TAMs through the NF-κB pathway and shifts the cytokine profiles within TME towards an antitumor response. This alteration reinstates the T cell reactivity towards the anti-PD-1 blockade (35, 73). The *Lm*-GP61 vaccine developed using the CD4^+^ T cell epitopes can impede tumor progression by inducing T_H_1 and CTL responses, synergizing PD-L1 blockade to mediate tumor suppression (83). In a mouse melanoma model, ICI synergized with an *Lm*-based melanoma vaccine to induce protective primary and memory T-cell responses through antigen-specific CD8^+^ T cells (62).

Nevertheless, the clinical utility of ICI remains restricted due to unresolved obstacles. Owing to tumor heterogeneity, a part of patients with responsive forms of cancer even exhibit no response to current ICI (84). Moreover, in spite of the strong association between immune checkpoint molecule expression and tumor progression, the lack of predictive biomarkers narrows down the benefit population. And it is controversial to use immune checkpoint molecule as the only predictive biomarkers for tumor immunotherapy (85). Hence, in view of this problem, some studies found other factors such as a T cell inflamed gene-expression profile (GEP) and tumor mutational burden (TMB) that can now be used as biomarkers to predict the responsiveness of ICI therapy (86).

### 4.2 Reenergized adoptive cell therapy

Adoptive cell therapy (ACT) using naturally or engineered immune cells has unprecedented success in oncotherapy, such as hematopoietic malignancies and melanoma (87–89). ACT is dependent on an adequate quantity of anti-tumor T cells with the requisite capabilities to cause cancer regression (90). Thus, the immunosuppressive TME may become an obstacle to progress in enhancing the efficacy of ACT (91). Considering the ability of *Lm*-based vaccines to infect and deplete MDSCs in TME, it may be the viable option that overcomes the limitations and enhances the efficacy of ACT (92).

Xin et al. developed a new strategy called Reenergized ACT (ReACT) which combines ACT with *Lm*-based vaccine, integrating the advantages of two methods (64). The bacteria/tumor-(dual) specific T cell is tumor-reactive CD8^+^ T cell that has been assembled *in vitro* with an extra T-cell receptor against a bacterial antigen. And the successful eradication of tumors relies on the substantial T cells within the tumor. The use of *Lm*-based vaccine leads to a significant reduction of MDSCs in TME to increase the intensity of specific T cell action, which makes the dual- specific T cells expand and migrate to the tumor area (64). The study in rodents demonstrated that ReACT enhanced the infiltration and function of CD8^+^ T cells in tumors, as well as reduced the expression of immune checkpoint molecules (93). In preclinical cancer models, ReACT has exhibited a fascinating efficacy in eradicating the primary tumor and reducing recurrences in the long term (93).

Despite the powerful effects, ReACT still faces some limitations. Local injection, which is used to ensure access to tumor tissues, may restrict the clinical application. Meanwhile, ReACT-induced immune responses may target self-antigens, resulting in normal cell and tissue injury. These concerns may become an important area of experimental and clinical investigation in the years to come.

### 4.3 Radiotherapy

Radiotherapy (RT) was shown to increase susceptibility of tumor cells to active vaccine therapy. Sublethal irradiation promoted the recruitment of infiltrating T cells in the treatment sites. The phenotype of tumor cells can be changed and more sensitive to *Lm*-based therapy (94, 95). Given the anti-tumor effects of radiation and *Lm*-based vaccine, these two methods can better control tumor development (63). Different immune responses elicited by different treatments have a synergistic effect. Combination of RT and chicken ovalbumin (OVA)-expressing Δ*actA*/Δ*inlB Lm* (*Lm*-OVA) increased the number of activated T cells in tumor tissues compared with either alone (63). Similarly, the combined administration of RT and *Lm*-based prostate cancer vaccine (ADXS31-142) accelerates tumor regression through enhanced specific immune responses in the prostate (68). Through coupling ^188^Rhenium and attenuated (at) *Lm* (*Listeria* ^at^), Quispe-Tintaya et al. created a unique radioactive *Listeria* ^at^ (RL) (96). RL delivered a high level of radioactive payload to metastatic colonies causing malignant cell death without normal tissue damage, holding great promise for controlling metastases. However, further clinical trials are still needed to determine their efficacy.

## 5 Application of *Lm*-based vaccine in solid tumors

*Lm*-based vaccines have been tested in many preclinical and clinical trials for different tumors, including cervical cancer, melanoma, pancreatic cancer, breast cancer, prostate cancer, and malignant pleural mesothelioma (**Tables 1**, **2**).

**Table 2:** Clinical trials of *Lm*-based vaccines.

NCT No.	Therapy (*Lm* vaccine with or without combination therapy)	cancer	Endpoints	Reference
NCT02325557	ADXS31142	mCRPC	ORR, PFS, OS, and immunogenicity	(67)
NCT03371381	JNJ-809	mCRPC	RP2D	(69)
NCT02592967	JNJ-757	NSCLC	immunogenicity, safety, efficacy	(97)
NCT01675765	CRS-207	MPM	Safety, induction of immune response	(70)
NCT02243371	CRS-207+ Cy/GVAX+ nivolumab	PDAC	OS, ORR, PFS, safety, tumor marker kinetics, immunologic responses	(71, 72)
NCT01417000.	CRS-207+ Cy/GVAX	PDAC	OS, PFS, OR	(98, 99)

The table summarizes clinical trials of *Lm*-based vaccines covered in the text.

### 5.1 HPV-associated cervical cancer

Chronic infection with HPV, especially type 16, is the main risk factor for the development of cervical cancer, the fourth most common cancer in women (100). The efficacy of current therapeutic interventions still acquires extensive substantiation (101). Due to the poor prognosis, there remains a lack of consensus regarding the second-line alternatives (102).

Axalimogen filolisbac (ADXS11-001), a new vaccine based on living attenuated *Lm*, is consisted with LLO and HPV-16 E7 antigen. ADXS11-001 can activate special immune responses to the E7-expressing malignant cells (58). It also increases the number of tumor infiltrating lymphocyte (TIL) and alleviates the immunosuppression status of TME (103). The results obtained from phase I/II/III trials encourage future perspectives for cervical cancer patients (58).

Accumulating evidence confirms that the combination of two different recombinant *Lm* strains exhibits a more satisfactory anti-tumor potential than using them alone. Treating HPV-infected mice with LMΔ*actA*plcB-E6E7 (LMΔ-E6E7) and LIΔ*actA*plcB-E6E7 (LIΔ-E6E7) can significantly overcome anti-vector immunity and accelerate the regression of tumor than the effect of LIΔ−E6E7 (11). In addition, some studies demonstrated that the optimization of codon usage contributes to the improvement of host immunity against TAAs (104, 105). The codon-optimized LM4Δ*hly*::E7-1 induced stronger Th1-biased immunity, lymphocyte proliferation, and specific CTL activity compared with LM4Δ*hly*::E7. Moreover, LM4Δ*hly*::E7 exhibited a significant improvement in the efficacy of tumor establishment treatment (59).

### 5.2 Melanoma

Melanoma has been proposed as the most aggressive type of skin cancer originating from melanocytes. Attenuated Δ*actA*/Δ*inlB Lm*, capable of expressingmelanoma inhibitory activity (MIA), can inhibit the growth of melanoma through the downregulation of blood vessel density (106). The human high molecular weight melanoma-associated antigen (HMW-MAA)-expressing *Lm* (*Lm*-LLO-HMW-MAA-C) acquires the ability to induce cell-mediated immune responses against HMW-MAA, targeting both tumor cells and pericytes in the tumor vascular system (60).

Non-targeting *Lm* still demonstrates the ability to induce melanoma cell death without specific target antigen. *Lm* ^at^-LLO, which produces ROS and causes a wide range of melanoma cell apoptosis, significantly reduced the size, volume, and metastatic burden of melanoma in Braf/Pten genetically engineered mice (61). The OVA-expressing *Lm* with the deletion of actA and phospholipase C stimulated strong CD8^+^ T cell responses including activation of both primary and memory T cells, resulting in protection against melanoma in mouse model transplanted with B16F10 cell line (62). Moreover, in combination with ICI treatment or RT, *Lm* vaccine increases the infiltration of antigen-specific CD8^+^ T cell and NK cells and shows better effects on reducing tumor size (62, 63).

### 5.3 Pancreatic cancer

Despite the recent advances in diagnosis and treatment, PDAC is still the fourth contributor to deaths from cancer (107). Gemcitabine (GEM) and erlotinib add up to six months to the median survival of patients with advanced PDAC (108, 109). It is difficult for many therapeutics to reach the tumor site due to the stromal barrier of PDAC (110, 111). With the ability to use MDSC as a vector, *Lm* could penetrate the primary tumor, which is considered to be a more efficient anti-tumor strategy (96).

*Lm*-expressing mesothelin (CRS-207) is of great clinical importance for *Lm*-based PDAC treatment. The administration of low-dose cyclophosphamides (Cy) prior to GM-CSF-secreting allogeneic pancreatic tumor cells (GVAX) (Cy/GVAX) followed by CRS-207 leads to a significant improvement in outcomes in a randomized multicenter phase II study with manageable toxicity (98, 99, 112). However, this strategy (Cy/GVAX + CRS-207) failed to improve the overall survival in a randomized phase IIB study compared to chemotherapy (113). This apparent paradox might be explained by the fact that nontargeted immunosuppressive vaccines are quite difficult to obtain ideal anti-tumor effects. Thus, current clinical trials focus on the combination of the prime-boost vaccination strategy and immune checkpoint inhibitors. Although these issues are a matter of debate, the current study using PD-1 inhibitor (nivolumab) + Cy/GVAX + CRS-207 yielded valuable insights into this field (71, 72).

Immunogenic tetanus toxoid protein (TT(856–1313)) can be delivered directly into PDAC through *Lm* vaccine and kill infected tumor cells via reactivating previously existing TT-specific memory T cells. Through intraperitoneal injection, *Lm* directly infects MDSCs which will migrate into TME, leading to the dissemination of *Lm*. Moreover, *Listeria*-TT regulates the number and function of macrophages and MDSCs, thus enhancing the sensitivity of tumor to GEM (40). At the same time, it avoids the side effects accompanying the use of high-dose GEM due to reduced sensitivity (40).

Selvanesan et al. developed an attenuated non-toxic and non-pathogenic *Listeria*-^32^P as a novel delivery platform, which kills tumor cells by ^32^P-induced ionizing radiation and *Lm*-induced ROS (114). The treatment causes a reduction in the growth of PDAC in KPC (conditionally express endogenous Kras-G12D, p53-R172H and pdx1-Cre mutant alleles) mice. Meanwhile, the radioactive *Lm* can precisely pinpoint the metastatic lesions in distant organs with minimal side effects to the adjacent normal tissue (114).

Besides, the combination of *Lm*-based immunotherapy with other treatments, such as radiotherapy, shows a promising anti-tumor effect and has a clinical future to prevent recurrence and metastasis of pancreatic cancer (96).

### 5.4 Breast cancer

Breast cancer (BC) is one of the most common malignancies in women. More than 20% of patients died of metastatic lesions and intervention resistance (115, 116). First-line strategy for metastatic cancer so far is surgery followed by chemotherapy or radiation (117). Despite the recent advances in BC therapy, the elimination of metastatic or primary tumor cells after initial treatment is often incomplete (118). More aggressive strategies are required, but few options are available, resulting in an urgent need for other effective measures. Immunotherapy has shown a promising perspective and can be served as an important candidate for BC patients.

The administration of Mage-b cDNA-expressing *Lm* (LM-LLO-Mage-b/2nd) before the establishment of tumor shows a more effective function of eliminating metastases than that of *Lm*-LLO in 4T1 BC model (65). The TAA Mage-b-expressing *Lm* (*Lm*-Mage-b) is combined with some immunologic adjuvants to promote a strong immune response. *Lm*-Mage-b can synergize with α-galactosylceramide and contribute to the increase of NK T cells in the spleen and the elimination of metastatic colonies without cellular toxicity (66). Triple-negative BC (TNBC) represents an extremely aggressive subtype that lacks estrogen receptor, progesterone receptor, and human epidermal growth factor receptor 2 (HER-2) (119, 120). Curcumin inhibits the production of MDSCs-derived IL-6 and enhances the effects of *Lm*-Mage-b through strong CD8^+^ T cell responses, resulting in a higher level of efficacy against metastases (121). *Listeria* ^at^ can decrease the number of infected MDSCs in blood and primary tumors. IL-12 is one of the signals to stimulate clonal expansion of CD8^+^ T cells (122).

### 5.5 Prostate cancer

It is estimated that 288,300 new cases of prostate cancer and 34,700 death cases will occur in 2023 (1). Up to 20% of men diagnosed with prostate cancer in the United States have regional or metastatic disease (123). Most of these patients who are treated with 1-3 years androgen-deprivation therapy will finally develop metastatic castration-resistant prostate cancer (124).

PSA can be detected in the majority of prostate cancer cases and has been identified as the target antigen (25). ADXS31-142, a live attenuated *Lm*-based immunotherapy, is bioengineered to secrete a fusion protein composed of truncated fragments of *Lm* lysozyme toxin (tLLO) and PSA, termed tLLO-PSA (67). A study demonstrated that the combination of ADXS31-142 and pembrolizumab is safe and well tolerated in patients with metastatic castration-resistant prostate cancer (67).

JNJ-64041809 (JNJ-809), a newly developed immunotherapy based on Δ*actA*/Δ*inlB Lm*, targets four prostate cancer antigens, including prostatic acid phosphatase (125), prostate-specific membrane antigen (126), synovial sarcoma X breakpoint 2 (127), and homeobox protein NKX3.1 (128). After the evaluation, the safety of JNJ-809 is manageable and the interventions implemented at an early stage may induce a more intense response. However, the observable antigen-specific immune response is limited and did not translate into an objective clinical response (69).

### 5.6 Malignant pleural mesothelioma

Exposure to asbestos or other small carcinogenic fibers is most likely responsible for malignant pleural mesothelioma (MPM), a rare malignancy with a poor prognosis (129). Although the combination of pemetrexed and cisplatin has been applied as standard initial treatment for patients with unresectable MPM (130), the high mortality has been promoting the search of alternative treatments (70). Accumulating evidence suggests that immunotherapeutic approaches can be used as promising treatments for MPM (131–134).

Almost all epithelial MPMs overexpress mesothelin (120, 135–137). When combined with chemotherapy, CRS-207 increases infiltration of dendritic NK cells and T cells, accompanied by a transition of macrophage from immunosuppressive M2 to proinflammatory M1 (70). In a phase Ib study in MPM, CRS-207 in combination with pemetrexed/cisplatin increases CD8^+^ T cell ratio and the infiltration of DCs and NK cells. The therapy leads to a notable reduction in tumor size without serious treatment-associated side effects (70). Besides, cytoreduction surgery could reduce the immunosuppression to restore the mesothelin-expressing *Lm* vaccine efficacy (138).

## 6 Future perspectives

By overcoming the immunosuppressive microenvironment and enhancing targeted anti-tumor immune responses, *Lm* vaccines have shown promising performance in the treatment of both primary and metastatic tumors.

However, the side effects of *Lm* vaccines are similar to other classical immunotherapies, including potential systemic immune response, hypertension, and fatigue. As a pathogen, *Lm* has raised concerns about its safety risks, even bacteremia. Several studies have explored the delicate balance between the efficacy and safety of *Lm*-based vaccines (69, 97, 99, 139, 140). At the same time, a lack of technical facilities limits the ability to test whether a sufficient number of vaccines can reach the tumor site.

Therefore, future research will focus on the development of less toxic and more potent *Lm* strains. At present, this target is mainly achieved by deleting virulence factors or developing KBMA and *Lm*-RIID strain. More effective evaluation methods are generally expected to select the best candidates among developed strains. In addition, several studies have explored the combined use of *Lm*-based vaccines with ICI, ReACT cells, radiotherapy, and other therapies, achieving remarkable results. The ability of *Lm*-based vaccine to reshape TME and enhance the immune response of CD8^+^ T cells endows it to exert synergistic anti-tumor effects. Meanwhile, how the *Lm*-based vaccine helps other approaches to regulate the immune status of TME may become an important area of clinical investigation in years to come.

The differences of immune system between human and experimental species remain a big obstacle to progress in developing *Lm-*based vaccines. Robust CD8^+^ T cell immunity can be elicited by *Lm*-based vaccine to specifically target a tumor antigen in mouse model. However, this result has not been confirmed in human so far. Substantial evidence suggests the possibility that the differences of γδ T cells between mouse and human may contribute to this phenomenon. According to the expression of γδ receptors, T cells can be divided into two subtypes, including γδ T cells (γδ^+^) and αβ T cells (γδ^-^) (141). Vγ9Vδ2 T cells represent the majority of human γδ T cells while murine γδ T cells are largely Vγ5Vδ1^+^ (142, 143). Through upregulation of cholesterol metabolism, *Lm*-infected human dendritic cells are able to activate Vγ9Vδ2 T cells. And in contrast to mouse, *Lm* infection in humans induces a distinct proliferation of Vγ9Vδ2 T cells (144). Besides, colorectal cancer antigen guanylyl cyclase C (*Lm*-GUCY2C)-expressing *Lm*-based vaccine can induce strong *Lm*-specific immunity, rather than anti-GUCY2C response. This result indicates that the competition with immunodominant *Lm*-derived CD8^+^ T-cell epitope may be involved. Some weak antigens, such as GUCY2C, may exhibit susceptibility to the competition from *Lm*-derived peptides (145). Despite recent advances, the underlying mechanisms of *Lm*-derived peptides may explain the unresolved problem in the setting of different species. Further research is significant to explore the synergy between existed vaccines and other treatments.

## Author contributions

YD: Formal Analysis, Project administration, Writing – original draft, Writing – review & editing. LS: Investigation, Writing – review & editing. RH: Writing – review & editing. KC: Writing – review & editing. YD: Writing – review & editing. ZZ: Writing – review & editing. YX: Funding acquisition, Resources, Supervision, Validation, Writing – review & editing. HD: Funding acquisition, Resources, Supervision, Validation, Writing – review & editing.
